# CHCHD3(MIC19): mitochondrial cristae structure regulation and disease associations

**DOI:** 10.3389/fmolb.2026.1861303

**Published:** 2026-06-11

**Authors:** Zheng Tai Kuai, Xiao Min Zheng, Yue Yue Li, Tian Qi Wang

**Affiliations:** 1 Wuxi Maternity and Child Health Care Hospital, Affiliated Women’s Hospital of Jiangnan University, Wuxi, China; 2 Department of Clinical Medicine, Wuxi School of Medicine, Jiangnan University, Wuxi, China

**Keywords:** apoptosis, CHCHD3, energy metabolism, MIC19, MICOS complex, mitochondrial contact sites, mitochondrial cristae

## Abstract

Mitochondrial bioenergetic competence critically depends on cristae architecture, which is organized and stabilized by the mitochondrial contact site and cristae organizing system (MICOS) complex. As a core MICOS subunit, CHCHD3 (also known as MIC19) contributes to assembly of the mitochondrial intermembrane space bridging (MIB) supercomplex and regulates cristae morphology, endoplasmic reticulum–mitochondria contact sites, and cellular metabolic homeostasis. Aberrant CHCHD3 expression or functional deficiency is implicated in the pathogenesis of neurodegenerative disorders, cardiovascular diseases, metabolic syndromes, and cancers. Notably, CHCHD3 function is governed by a dose-dependent “Goldilocks” principle, wherein both insufficient and excessive expression—as well as preserved abundance with impaired functional integrity—can compromise mitochondrial homeostasis, underscoring the need for context-specific therapeutic modulation. Here, we systematically summarize CHCHD3 molecular characteristics and post-translational modification networks, with emphasis on its roles in energy metabolism, organelle crosstalk, and apoptosis. We further examine the mechanistic links between CHCHD3 dysregulation and disease pathogenesis, evaluate current targeting strategies and their pharmacological limitations, and identify remaining controversies and knowledge gaps to guide future research toward clinical translation.

## 1 Introduction

Mitochondria generate the majority of cellular ATP through oxidative phosphorylation and additionally govern apoptosis, calcium signaling, reactive oxygen species homeostasis, innate immune signaling, and metabolic reprogramming (Glover et al., 2024; Zhang et al., 2023). Consequently, mitochondrial dysfunction contributes to a broad spectrum of human diseases, including neurodegenerative disorders, cardiovascular diseases, metabolic syndromes, and cancer (Zong et al., 2024).

Recent advances in cryo-electron microscopy and super-resolution imaging have enabled detailed structural characterization of the mitochondrial contact site and cristae organizing system (MICOS) (Vue et al., 2023a; Frey and Mannella, 2000), a conserved multi-subunit complex enriched at crista junctions that stabilizes inner membrane architecture and preserves bioenergetic compartmentalization (Friedman et al., 2015; Harner et al., 2011; Eramo et al., 2020).

CHCHD3, also known as MIC19, is a core MICOS component containing a coiled-coil-helix–coiled-coil-helix domain. It is essential for MICOS assembly and stability, cristae maintenance, endoplasmic reticulum–mitochondria contact regulation, and cellular energy metabolism (Sakowska et al., 2015; Dong et al., 2024). Dysregulated CHCHD3 expression or impaired function is increasingly associated with the same spectrum of pathological conditions noted above, highlighting its potential as a disease biomarker and therapeutic target (Dong et al., 2024; Theocharopoulou, 2020; Vue et al., 2023b; Fujita et al., 2025).

In this review, we establish the structural framework of MICOS and its supramolecular assembly with the outer membrane sorting and assembly machinery (SAM), then systematically summarize the molecular features, post-translational modification networks, and mitochondrial import pathway of CHCHD3. We further discuss its multifaceted biological functions—spanning cristae morphology, organelle crosstalk, energy metabolism, and apoptosis—and examine the pathological relevance of its dysregulation across neurodegenerative, cardiovascular, oncological, and metabolic diseases, with particular attention to the dose-dependent “Goldilocks” principle governing its expression. Finally, we evaluate current therapeutic strategies and their pharmacological limitations, aiming to provide a comprehensive conceptual framework for understanding CHCHD3 biology and its translational potential.

## 2 The MICOS complex and mitochondrial cristae architecture

**FIGURE 1:**
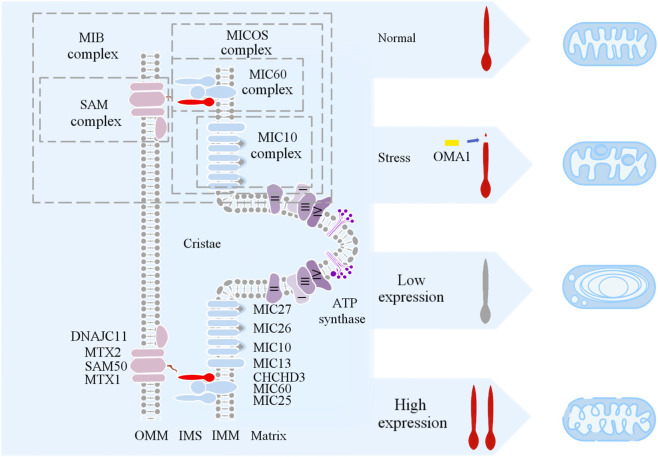
The CHCHD3-centered MICOS–MIB supercomplex and dose-dependent effects on mitochondrial cristae architecture (Left) Schematic of the mitochondrial intermembrane space bridging (MIB) supercomplex spanning the outer mitochondrial membrane (OMM) and inner mitochondrial membrane (IMM). The SAM complex (SAM50, MTX1, MTX2, DNAJC11) in the OMM associates with the MICOS complex (MIC60, MIC25, CHCHD3/MIC19, MIC10, MIC13, MIC26, MIC27) at the IMM via the CHCHD3 bridge. CHCHD3 engages SAM50 through its N-terminal region and MIC60 through its C-terminal CHCH domain, thereby stabilizing the SAM50–CHCHD3–MIC60 axis. ATP synthase dimers localize at cristae tips to promote membrane curvature (Right) Dose-dependent effects of CHCHD3 on cristae morphology under four conditions. Normal: physiological CHCHD3 levels maintain intact cristae junctions (CJs) and organized lamellar cristae. Stress: OMA1-mediated cleavage of CHCHD3 generates truncated S-CHCHD3 with impaired SAM50-binding capacity, leading to MIB destabilization and CJ loss. Low expression: CHCHD3 deficiency causes severe cristae junction loss and disorganized “onion-like” vesiculated cristae. High expression: supraphysiological CHCHD3 accumulation promotes cristae hyper-condensation and excessive ROS production. Abbreviations: OMM, outer mitochondrial membrane; IMS, intermembrane space; IMM, inner mitochondrial membrane; MIB, mitochondrial intermembrane space bridging; SAM, sorting and assembly machinery; MICOS, mitochondrial contact site and cristae organizing system; CJs, cristae junctions.

To contextualize the role of CHCHD3 in mitochondrial architecture, we first describe the structural organization of the mitochondrial contact site and cristae organizing system (MICOS) and its supramolecular assembly with the outer membrane sorting and assembly machinery (SAM) complex (Figure 1).

### 2.1 Mitochondrial ultrastructure and the discovery of MICOS

Mitochondria are bounded by the outer mitochondrial membrane (OMM) and inner mitochondrial membrane (IMM), which delineate the intermembrane space (IMS) and matrix. The IMM forms cristae, specialized membrane invaginations that connect to the peripheral IMS through narrow cristae junctions (CJs). These structures are essential for preserving inner membrane architecture and establishing bioenergetically distinct submitochondrial compartments (Herrmann and Riemer, 2010; Mannella, 2006; Pfanner et al., 2014). Cristae increase the membrane surface available for respiratory chain supercomplex assembly and oxidative phosphorylation, and also serve as signaling microdomains for metabolic control and apoptosis (Ježek et al., 2023; Rao et al., 2026).

The MICOS complex was first implicated in mitochondrial architecture with the discovery of mitofilin, currently known as MIC60, in 1994 (Funck, 2022). Its molecular composition and role in cristae organization were systematically characterized by independent studies in 2011 (Harner et al., 2011; Hoppins et al., 2011; von der Malsburg et al., 2011), followed by the introduction of a unified MICOS nomenclature in 2014 (Pfanner et al., 2014).

### 2.2 Composition and hierarchical assembly of the MICOS complex

In mammalian mitochondria, the canonical MICOS complex is composed of the core subunits MIC60, MIC19/CHCHD3, MIC10, MIC25/CHCHD6, MIC26, MIC27, and MIC13/QIL1. CHCHD10 has also been described as a MICOS-associated protein in certain cellular or experimental contexts; however, whether it should be considered a constitutive core component of the complex remains incompletely resolved (Eramo et al., 2020).

MICOS biogenesis follows a hierarchical assembly pathway. The MIC60–MIC19–MIC25 subcomplex is thought to form an early scaffold at the inner mitochondrial membrane, which subsequently supports incorporation of the MIC10–MIC26–MIC27 module involved in membrane remodeling. MIC13/QIL1 then acts as a bridging factor that stabilizes the association between these modules and promotes formation of the mature MICOS holo-complex (Sastri et al., 2017; Anand et al., 2016; Kondadi et al., 2020). Consistent with this model, structural analyses have shown that the CHCH domain of MIC19/CHCHD3 engages the mitofilin domain of MIC60, thereby stabilizing a bow-tie-shaped tetrameric architecture that contributes to MICOS anchoring at cristae junctions (Bock-Bierbaum et al., 2022).

### 2.3 The MIB supercomplex and the role of SAM50

MICOS physically and functionally interfaces with the outer mitochondrial membrane sorting and assembly machinery (SAM) complex to form the mitochondrial intermembrane space bridging (MIB) supercomplex. This transmembrane organizational platform connects the inner and outer mitochondrial membranes and contributes to the coordinated regulation of cristae architecture, membrane contact sites, and mitochondrial protein biogenesis (Huynen et al., 2016; Rampelt et al., 2018). The core SAM component SAM50 comprises N-terminal POTRA domains exposed to the intermembrane space (IMS) and a C-terminal β-barrel domain embedded within the outer mitochondrial membrane. Within this architecture, CHCHD3 serves as a molecular bridge between the two membranes: its N-terminal region interacts with the IMS-facing surface of SAM50, whereas its C-terminal CHCH domain binds the mitofilin domain of MIC60, thereby stabilizing the SAM50–CHCHD3–MIC60 axis (Tang et al., 2020a; Ott et al., 2012). This structural arrangement is further supported by cryo-electron microscopy analyses that define the interaction interface between the CHCH domain of MIC19/CHCHD3 and the mitofilin domain of MIC60 (Bock-Bierbaum et al., 2022).

Under mitochondrial stress conditions, the inner membrane metalloprotease OMA1 cleaves CHCHD3 within its N-terminal region, generating a truncated form, S-CHCHD3, with reduced capacity to bind SAM50. Loss of this interaction weakens the MIB bridge, perturbs cristae junction stability, and promotes defects in mitochondrial bioenergetic function (Tang et al., 2020b).

Together, these findings position CHCHD3 as a central architectural component that couples MICOS-dependent cristae scaffolding to MIB-mediated intermembrane bridging (Huynen et al., 2016). Accordingly, disruption of CHCHD3—through genetic depletion, dysregulated expression, or stress-induced proteolytic processing—impairs cristae organization and compromises mitochondrial bioenergetic competence (Bock-Bierbaum et al., 2022; Tang et al., 2020b; Vue et al., 2024a).

## 3 Molecular characteristics of CHCHD3

**FIGURE 2:**
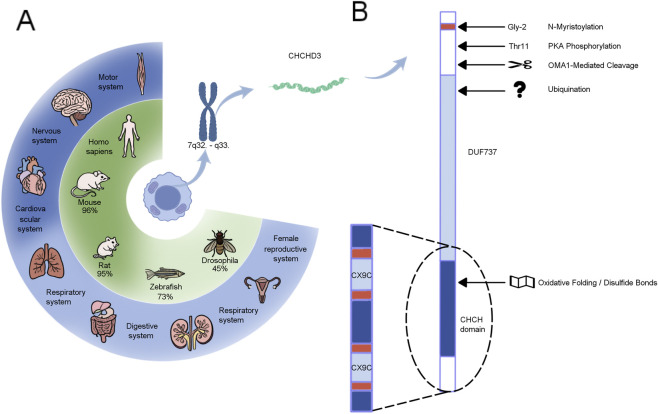
CHCHD3 genomic localization, protein architecture, tissue expression profile, and validated post-translational modification sites. **(A)** The human CHCHD3 gene resides at chromosome 7q32–q33 and is evolutionarily conserved across species (*Homo sapiens*, *Mus musculus*, *Rattus norvegicus*, *Danio rerio*, and *Drosophila*). CHCHD3 is ubiquitously expressed in multiple human organ systems, including the nervous, cardiovascular, respiratory, digestive, urinary, motor, and female reproductive systems. **(B)** Schematic of CHCHD3 domain architecture and validated PTM sites. The N-terminal segment contains a validated N-myristoylation site at Gly-2 (yellow), a PKA phosphorylation site at Thr11 (red), and the OMA1-mediated proteolytic cleavage region (scissors). The central DUF737 domain (residues ∼14–175) provides a conformational scaffold. The C-terminal CHCH domain (residues ∼176–217) contains two canonical CX9C motifs (Cys183–Cys193 and Cys204–Cys214) whose intramolecular disulfide bonds (yellow dashed lines) are introduced by the MIA40/CHCHD4 oxidative folding machinery in the intermembrane space. K48-linked ubiquitination by the E3 ligase ASB1 (green Ub) targets CHCHD3 for proteasomal degradation; the specific substrate lysine residue remains to be experimentally mapped (indicated by “?”). Abbreviations: CX9C, cysteine–X9–cysteine motif; Ub, ubiquitin.

To define the molecular basis by which CHCHD3 functions as a structural and regulatory hub, we next examine its gene organization, conserved domain architecture, expression pattern, and post-translational regulatory mechanisms (Figure 2).

### 3.1 Gene nomenclature and evolutionary conservation

The human CHCHD3 gene, originally annotated as FLJ20420, is located on chromosome 7q32.3–q33 and encodes a 227-amino-acid protein (Ota et al., 2004). CHCHD3 was initially identified by Schauble et al. as a candidate substrate of protein kinase A (PKA) and was subsequently assigned to the CHCHD protein family based on the presence of a C-terminal coiled-coil-helix–coiled-coil-helix (CHCH) domain containing two conserved CX_9_C motifs (Schauble et al., 2007; Darshi et al., 2011; Longen et al., 2009). Early co-immunoprecipitation studies revealed an association between CHCHD3 and mitofilin/MIC60 (Xie et al., 2007), whereas subsequent functional analyses by Darshi et al. demonstrated its mitochondrial localization and established its requirement for maintaining normal cristae morphology and respiratory competence (Darshi et al., 2012). Later systematic studies further defined CHCHD3, together with MIC60 and MIC25/CHCHD6, as a core component of the MIC60-containing assembly module that nucleates MICOS complex formation (Guarani et al., 2015).

CHCHD3 is broadly conserved across eukaryotes, with the human protein sharing approximately 96%, 95%, 73%, and 45% amino acid identity with its mouse, rat, zebrafish, and *Drosophila* orthologs, respectively. This conservation is particularly pronounced within the C-terminal CHCH domain, underscoring its functional importance in MICOS assembly and mitochondrial inner membrane organization (Darshi et al., 2011).

### 3.2 Structural characteristics of CHCHD3 protein

Human CHCHD3 comprises three major structural regions: an N-terminal segment containing a predicted N-myristoylation motif at Gly-2, a central domain of unknown function 737 (DUF737; approximately residues 14–175), and a C-terminal coiled-coil-helix–coiled-coil-helix (CHCH) domain spanning approximately residues 176–217 (Darshi et al., 2012; Kozjak-Pavlovic, 2017).

The N-terminal myristoylation motif has been proposed to facilitate mitochondrial membrane association and incorporation of CHCHD3 into MICOS- and MIB-related assemblies. However, this motif is unlikely to act as an autonomous targeting determinant and probably functions in concert with CHCH domain-dependent mitochondrial import, oxidative folding, and intramitochondrial retention mechanisms (Darshi et al., 2012; Utsumi et al., 2018). The intervening DUF737 region may provide a structural or protein-interaction platform that couples the N-terminal membrane-association region to the C-terminal CHCH domain, although its precise contribution to CHCHD3 stability, assembly, and regulation remains insufficiently defined. Notably, the N-terminal region contains an experimentally validated PKA phosphorylation site at Thr11, whereas the potential existence and functional relevance of additional phosphorylation sites within DUF737 require further investigation (Schauble et al., 2007).

The C-terminal CHCH domain contains two canonical CX_9_C motifs, Cys183-X_9_-Cys193 and Cys204-X_9_-Cys214, which form intramolecular disulfide bonds that stabilize a characteristic helix–loop–helix conformation (Longen et al., 2009). This domain constitutes a principal interaction interface for MIC60/mitofilin, supporting MIC60 oligomerization and contributing to the stabilization of MICOS architecture at cristae junctions (Dong et al., 2024; Bock-Bierbaum et al., 2022).

### 3.3 Tissue expression and subcellular localization

CHCHD3 is widely expressed across mammalian tissues, with relatively high abundance in organs characterized by elevated oxidative and energetic demand, including the heart, skeletal muscle, liver, kidney, and brain (Dong et al., 2024; Sohn et al., 2023). At the subcellular level, CHCHD3 localizes predominantly to mitochondria, where it is enriched at or near cristae junctions as a component of MICOS-associated structures. Nevertheless, its precise submitochondrial distribution may vary according to cell type, metabolic state, and mitochondrial remodeling context (Sastri et al., 2017; Darshi et al., 2011; Darshi et al., 2012).

In addition to its mitochondrial pool, low-abundance extramitochondrial CHCHD3 has been reported in both the cytoplasm and nucleus. One study suggested that nuclear CHCHD3 may associate with the BAG-1 promoter and modulate apoptosis-related signaling; however, the mechanism governing nuclear entry, the regulatory conditions that support this localization, and the broader physiological relevance of this proposed function remain to be established (Darshi et al., 2012; Liu et al., 2012).

### 3.4 Post-translational regulation of CHCHD3

The abundance, submitochondrial localization, and assembly competence of CHCHD3 are regulated by multiple post-translational mechanisms, including lipid modification, oxidative folding, phosphorylation, ubiquitin-dependent turnover, and stress-induced proteolytic processing. These modifications collectively influence the incorporation of CHCHD3 into MICOS- and MIB-associated complexes and thereby modulate mitochondrial membrane architecture and function (Schauble et al., 2007; Darshi et al., 2012; Zhao et al., 2024).

N-terminal myristoylation. After removal of the initiator methionine, Gly-2 of CHCHD3 is predicted to undergo N-myristoylation, most likely catalyzed by N-myristoyltransferase 1. This lipid modification may enhance membrane affinity and facilitate the stable association of CHCHD3 with MICOS/MIB assemblies (Darshi et al., 2012; Utsumi et al., 2018). Consistent with this notion, myristoylation-deficient CHCHD3 mutants show defective mitochondrial localization and are associated with cristae disorganization. Nevertheless, CHCHD3 mitochondrial targeting and assembly are unlikely to be determined by myristoylation alone; rather, they probably require the coordinated action of N-terminal membrane-association elements, CHCH domain-dependent import, and intramitochondrial retention mechanisms.

Oxidative folding. The conserved cysteine residues within the C-terminal CHCH domain form intramolecular disulfide bonds in the mitochondrial intermembrane space. These disulfide bonds are required to stabilize the characteristic CHCH fold and to support productive interaction with MIC60/mitofilin (Sakowska et al., 2015; Darshi et al., 2011; Darshi et al., 2012; Mukherjee et al., 2021). Thus, oxidative folding is a critical determinant of CHCHD3 maturation, structural stability, and incorporation into MICOS.

Phosphorylation. CHCHD3 was originally identified as a candidate PKA substrate, and Thr11 has been experimentally validated as a phosphorylation site using a phospho-specific antibody (Schauble et al., 2007). Phosphorylation at Thr11 has been reported to suppress Parkin recruitment to damaged mitochondria and to inhibit MICOS complex assembly (Akabane et al., 2016), suggesting that this modification may link kinase signaling to mitochondrial quality control and cristae remodeling. However, the mechanistic consequences of Thr11 phosphorylation remain incompletely resolved, particularly with respect to CHCHD3 conformation, MIC60 binding, MICOS incorporation, and cristae junction stability. Additional phosphorylation events, including candidate sites within the DUF737 region identified in phosphoproteomic datasets, require targeted biochemical and functional validation.

Ubiquitination. CHCHD3 turnover is regulated, at least in part, by ubiquitin-dependent proteasomal degradation. The E3 ubiquitin ligase ASB1 promotes K48-linked polyubiquitination of CHCHD3, thereby targeting it for degradation by the proteasome (Zhao et al., 2024). In prostate cancer models, reduced ASB1 expression leads to CHCHD3 accumulation, enhanced mitochondrial activity, and increased tumor cell proliferation, indicating that impaired CHCHD3 turnover may contribute to pathological metabolic remodeling and tumor progression (Zhao et al., 2024; Gan et al., 2025).

Stress-induced proteolytic processing. Under mitochondrial stress conditions, the inner membrane metalloprotease OMA1 cleaves CHCHD3 near its N terminus, generating a truncated species termed S-CHCHD3. This processed form exhibits reduced SAM50-binding capacity, thereby weakening the SAM50–CHCHD3–MIC60 axis and compromising MIB integrity (Tang et al., 2020a). As a consequence, OMA1-dependent CHCHD3 cleavage promotes cristae disorganization and may contribute to mitochondrial dysfunction during stress.

Post-translational modifications of CHCHD3 may not function independently, but could instead contribute to a broader regulatory framework influencing its maturation, assembly, and turnover. In this context, N-terminal myristoylation has been implicated in membrane association, oxidative folding of the CHCH domain is likely important for structural maturation and mitochondrial retention, and phosphorylation, ubiquitination, and stress-induced proteolysis may further modulate CHCHD3 stability or complex incorporation. It is possible that Thr11 phosphorylation affects CHCHD3 conformation or interaction properties, whereas correct disulfide-bond formation may be required for efficient MIC60 association and MICOS assembly. Likewise, CHCHD3 species that fail to assemble properly may be more susceptible to stress-induced cleavage or ubiquitin-dependent turnover, while stable incorporation into higher-order complexes could confer protection. Although direct evidence for such crosstalk remains limited, these observations collectively suggest that CHCHD3 may be subject to multilayered regulation, consistent with its proposed role in coordinating mitochondrial cristae architecture and membrane-contact-site homeostasis.

## 4 Mitochondrial import and hierarchical assembly of CHCHD3 into the MICOS and MIB supercomplex

**FIGURE 3:**
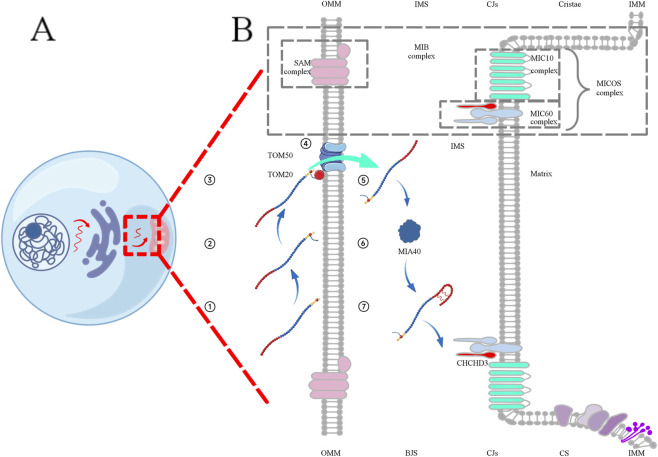
Detailed stepwise mechanism of CHCHD3 mitochondrial import and MICOS assembly. **(A)** Schematic overview of the cellular context, showing that CHCHD3 is encoded by the nuclear genome, translated in the cytoplasm, and targeted to mitochondria for functional maturation. **(B)** Detailed molecular pathway of CHCHD3 biogenesis and assembly (steps 1–7): (1) nuclear transcription and cytoplasmic translation of CHCHD3 precursor protein; (2) targeting of the newly synthesized CHCHD3 polypeptide to the mitochondrial outer membrane (OMM); (3) recognition of the precursor by the TOM complex receptors (TOM20, TOM50); (4) translocation of the unfolded precursor across the OMM through the TOM channel; (5) release of the precursor into the intermembrane space (IMS); (6) oxidative folding of CHCHD3 mediated by the MIA40 disulfide relay system, which introduces disulfide bonds to stabilize the protein structure; (7) assembly of correctly folded CHCHD3 into the MICOS (mitochondrial contact site and cristae organizing system) complex at cristae junctions (CJs). The MICOS complex consists of two subcomplexes: the MIC10 complex and the MIC60 complex, and interacts with the MIB complex and SAM complex on the OMM. The assembled MICOS complex maintains cristae architecture and anchors the respiratory chain complexes and ATP synthase on the inner mitochondrial membrane (IMM). Abbreviations: OMM, outer mitochondrial membrane; IMS, intermembrane space; CJs, cristae junctions; IMM, inner mitochondrial membrane; TOM, translocase of the outer membrane; MIA, mitochondrial intermembrane space assembly; MICOS, mitochondrial contact site and cristae organizing system; SAM, sorting and assembly machinery; MIB, mitochondrial import and biogenesis complex.

This section reviews the sequential molecular events that govern the mitochondrial import, oxidative folding, and hierarchical assembly of newly synthesized CHCHD3 into the MICOS complex and the MIB supercomplex (Figure 3).

### 4.1 Mitochondrial targeting and import of CHCHD3

Unlike presequence-containing mitochondrial matrix or inner membrane proteins that are imported predominantly *via* the canonical TOM–TIM23 pathway (Jain et al., 2025), CHCHD3 belongs to the class of cysteine-rich intermembrane space (IMS) proteins associated with the MIA pathway (Dickson-Murray et al., 2021). This pathway, centered on the oxidoreductase MIA40/CHCHD4, mediates the import and oxidative folding of small cysteine-containing IMS proteins (Al-Habib and Ashcroft, 2021). Consistent with this classification, CHCHD3 contains a C-terminal CHCH domain with two CX9C motifs (Ueda et al., 2019).

Notably, CHCHD3 import appears to differ in some respects from the canonical MIA-dependent import of other IMS proteins (van der Schans et al., 2026). Several studies indicate that efficient CHCHD3 import depends, at least in part, on TOM20, supporting a TOM20-facilitated or TOM20-dependent MIA import route (Darshi et al., 2012). In this model, newly synthesized CHCHD3 is initially recognized at the outer mitochondrial membrane through its N-terminal region, potentially aided by Gly-2 myristoylation. Rather than directly mediating TOM20 binding, this lipid modification may enhance mitochondrial membrane association and promote productive engagement with the TOM machinery (Schauble et al., 2007; Darshi et al., 2012). CHCHD3 is then transferred through the TOM40 channel into the IMS, where it gains access to the oxidative folding machinery (Sakowska et al., 2015). Although recognition and translocation through TOM components are likely important determinants of import efficiency, the kinetic basis of this process remains incompletely understood (van der Schans et al., 2026).

Earlier studies proposed TOM70 as a major import receptor for CHCHD3 and suggested that a PERK–OGT–TOM70 signaling axis regulates import through O-GlcNAcylation of TOM70 at Ser94 (Latorre-Muro et al., 2021). However, subsequent genetic depletion or knockout experiments, co-immunoprecipitation analyses, and *in vitro* import assays challenged this model and instead supported a predominant role for TOM20 in CHCHD3 import (Marada et al., 2024). These findings also raised doubts as to whether TOM70 functions as a relevant CK2-regulated receptor in this context (Marada et al., 2024). Thus, while current evidence favors TOM20-facilitated import, the broader mechanisms regulating CHCHD3 import under physiological and stress conditions remain to be defined (van der Schans et al., 2026).

### 4.2 Oxidative folding and retention in the intermembrane space

Following translocation into the IMS, CHCHD3 is captured by the MIA40/CHCHD4 disulfide relay system (Al-Habib and Ashcroft, 2021). Cys193 within the CHCH domain plays a critical role in its recognition by MIA40, thereby initiating the oxidative folding process that converts the import-competent precursor into a mature IMS protein capable of stable retention and subsequent assembly into the MICOS complex (Darshi et al., 2012). Importantly, CHCHD3 is not a classical multispanning inner membrane protein. Rather, the mature protein resides within the IMS and becomes enriched at crista junctions through its interactions with inner membrane-associated MICOS components, particularly MIC60 and MIC25/CHCHD6 (Yang et al., 2021). Oxidative folding and complex assembly are therefore functionally coupled processes that jointly determine the submitochondrial localization and stability of CHCHD3 (Darshi et al., 2012).

### 4.3 Hierarchical incorporation of CHCHD3 into the MICOS complex

Following oxidative maturation, CHCHD3 is incorporated into the MICOS complex through a hierarchical assembly pathway (Ott et al., 2015; van der Laan et al., 2016). Available evidence indicates that CHCHD3 first associates with MIC60 and MIC25/CHCHD6, thereby contributing to the formation or stabilization of a MIC60-containing core assembly module at crista junctions (Anand et al., 2016; Guarani et al., 2015; Stephan et al., 2020). Structural studies further suggest that the CHCH domain of CHCHD3 engages the mitofilin domain of MIC60, an interaction that supports tetrameric organization of the scaffold (Bock-Bierbaum et al., 2022). Subsequently, recruitment of the membrane-remodeling MIC10–MIC26–MIC27 subcomplex, bridged by MIC13/QIL1, promotes formation of the fully assembled MICOS holo-complex (Anand et al., 2016; Rampelt et al., 2018; Guarani et al., 2015). Disruption of CHCHD3 expression or maturation weakens this hierarchical assembly process, impairs recruitment or stabilization of downstream subunits, and ultimately leads to crista disorganization, mitochondrial fragmentation, and defective respiratory function (Darshi et al., 2011; Ott et al., 2015).

### 4.4 Assembly of the SAM50–CHCHD3–MIC60 axis and the MIB supercomplex

In addition to its role within MICOS, CHCHD3 contributes to the formation and stability of the mitochondrial intermembrane space bridging (MIB) supercomplex by linking the outer and inner mitochondrial membranes (Ott et al., 2012). SAM50, the central β-barrel component of the sorting and assembly machinery (SAM) complex, contains N-terminal POTRA domains exposed to the IMS (Ravi et al., 2025). CHCHD3 interacts with this IMS-accessible region of SAM50 through its N-terminal/DUF737 region, while simultaneously binding MIC60 through its C-terminal CHCH domain, thereby establishing the SAM50–CHCHD3–MIC60 bridging axis (Darshi et al., 2012). Cryo-electron microscopy studies support the structural compatibility of this CHCH–mitofilin interaction with higher-order MIB assembly (Bock-Bierbaum et al., 2022). This bridging axis represents a major determinant of MIB stability, although it is best understood as part of a broader membrane contact site network rather than as the sole structural element maintaining outer–inner membrane connectivity (Yin et al., 2022). Accordingly, disruption of any major component within this axis can compromise the integrity of the intermembrane bridging architecture (Tang et al., 2020b).

### 4.5 Stress-responsive remodeling of CHCHD3 assembly

The incorporation of CHCHD3 into MICOS and MIB is dynamically regulated rather than constitutive (Gilkerson et al., 2021). Under conditions of mitochondrial stress, the inner membrane metalloprotease OMA1 cleaves CHCHD3 within or near its N-terminal region, generating a truncated species termed S-CHCHD3 that is defective in SAM50 binding (Tang et al., 2020a). This proteolytic event weakens the SAM50–CHCHD3–MIC60 axis, destabilizes the MIB supercomplex, and promotes crista remodeling as part of a broader mitochondrial stress response that also includes OPA1 processing and dissipation of membrane potential (Tang et al., 2020a; Baker et al., 2014). Although CHCHD3 cleavage is clearly associated with MIB destabilization and bioenergetic failure, the precise temporal and mechanistic sequence linking these events remains to be fully elucidated.

In summary, CHCHD3 biogenesis proceeds through a coordinated sequence of TOM20-dependent import, MIA40-mediated oxidative folding, hierarchical incorporation into the MIC60–MIC25 scaffold, and subsequent engagement in the SAM50–CHCHD3–MIC60 bridging axis (Darshi et al., 2012; Wang et al., 2025). Under stress conditions, OMA1-dependent proteolysis disrupts this assembly pathway, thereby promoting rapid remodeling of mitochondrial architecture. Figure 2 illustrates the stepwise biogenesis of CHCHD3, from nuclear expression to oxidative maturation and assembly into the MICOS and MIB complexes (Tang et al., 2020a).

## 5 Biological functions of CHCHD3

This section delineates the functional roles of CHCHD3 across three interrelated processes: the maintenance of mitochondrial cristae architecture, the coordination of metabolic adaptation, and the regulation of apoptotic signaling.

### 5.1 Regulation of mitochondrial cristae architecture

Under physiological conditions, CHCHD3 appears to play an important role in maintaining cristae junction integrity through its association with the MIC60-containing MICOS complex and the SAM50–CHCHD3–MIC60 mitochondrial intermembrane space bridging (MIB) complex (Darshi et al., 2011; Darshi et al., 2012; Li et al., 2016). Evidence from loss-of-function studies indicates that CHCHD3 deficiency is associated with substantial disruption of cristae junctions, the formation of disorganized “onion-like” cristae structures, impaired respiratory-chain supercomplex assembly, reduced ATP production, and increased oxidative stress (Darshi et al., 2011; Li et al., 2016; Javadov et al., 2021; Bruni, 2021). Consistent with this structural role, mutational analyses of conserved cysteine residues within the CHCH domain suggest that preservation of the direct CHCHD3–MIC60 interaction alone may not be sufficient to maintain overall MICOS integrity, as recruitment of other MICOS components can still be compromised under these conditions (Darshi et al., 2012). Taken together, these findings support the view that CHCHD3 contributes to inner mitochondrial membrane organization and to the preservation of cristae architecture required for efficient oxidative phosphorylation (Darshi et al., 2011; Eydt et al., 2017; Anand et al., 2021). However, whether excessive stabilization of cristae structure might limit acute metabolic flexibility remains unclear (Resende et al., 2022; Smith et al., 2018).

### 5.2 Metabolic regulation through mitochondrial remodeling and ER–Mitochondria communication

CHCHD3 has emerged as a potential effector linking nutritional and hormonal signaling to mitochondrial remodeling, particularly in the liver (Darshi et al., 2011). Its fasting-induced upregulation supports fatty acid oxidation and oxidative phosphorylation by preserving cristae architecture and potentially at ER–mitochondria contact sites (Darshi et al., 2011; Sohn et al., 2023; Guo et al., 2013). An EMC2–SLC25A46–CHCHD3 axis has been proposed to mediate lipid transfer and inter-organelle communication, although its molecular organization and physiological generalizability remain unresolved (Dong et al., 2024).

Evidence from liver-specific knockout models further implicates CHCHD3 in hepatic metabolic homeostasis (Vue et al., 2024b). CHCHD3 deficiency is associated with cristae disruption, impaired respiration, dysregulated lipid metabolism, reduced ER–mitochondria contacts, and spontaneous MASH-like pathology, including steatosis, inflammation, and fibrosis, even in the absence of dietary stress (Vue et al., 2024b). Consistent with these findings, restoration of CHCHD3 expression by the FXR agonist INT-787 under metabolic stress is accompanied by improved mitochondrial ultrastructure and lipid homeostasis (Di Pasqua et al., 2024). Collectively, these observations position CHCHD3 as a candidate downstream mediator of metabolic signaling in liver physiology and disease (Darshi et al., 2011).

### 5.3 Regulation of apoptosis and stress responses

Within mitochondria, CHCHD3 contributes to the maintenance of cristae junction integrity, thereby preserving the sequestration of cytochrome c within cristae compartments (Darshi et al., 2011). Consistent with this role, CHCHD3 depletion facilitates cytochrome c mobilization and increases susceptibility to apoptosis, likely in conjunction with OPA1-dependent cristae remodeling, cardiolipin reorganization, and BAX/BAK-mediated outer membrane permeabilization (Gottschalk et al., 2022; Korytowski et al., 2011; Dudek, 2017).

In addition to its canonical mitochondrial localization, a minor nuclear pool of CHCHD3 has been reported (Liu et al., 2012). In one study, nuclear CHCHD3 was proposed to repress BAG-1 transcription, suggesting a potential role in modulating anti-apoptotic signaling (Liu et al., 2012). However, the mechanism underlying its nuclear localization and the extent to which this activity is conserved across cell types or stress conditions remain unclear. Possible explanations include stress-associated release from mitochondria, altered trafficking following proteolytic processing, or redistribution of a limited cytosolic pool when mitochondrial import is compromised (Baker et al., 2014; Weith et al., 2025). Given the lack of direct experimental validation, this nuclear function is best regarded as a context-dependent extension of CHCHD3 biology rather than an established general mechanism (Zhou et al., 2017).

### 5.4 Integrated view of CHCHD3 function

Collectively, current evidence supports CHCHD3 as a key determinant of mitochondrial inner membrane organization, with important implications for metabolic homeostasis and apoptotic susceptibility (Darshi et al., 2011; Darshi et al., 2012; Sohn et al., 2023). Its functional impact appears to be shaped by expression level, post-translational modification, subcellular localization, and cellular context, placing CHCHD3 at the interface of bioenergetic regulation, inter-organelle communication, and cellular stress responses (Oo et al., 2025; Zaccolo et al., 2021).

## 6 CHCHD3 and human diseases

Aberrant CHCHD3 expression or loss of functional integrity has been implicated in a broad spectrum of human diseases, including neurological disorders, cardiovascular disease, cancer, and metabolic dysfunction (Gerlach et al., 2025). Importantly, CHCHD3 dysfunction is not invariably accompanied by reduced total protein abundance (Yang et al., 2023). In several pathological settings, CHCHD3 levels are preserved or even elevated, whereas its function is compromised by stress-induced proteolytic processing. This impairment may also involve altered post-translational modification or destabilization of the SAM50–CHCHD3–MIC60 axis (Tang et al., 2020a; Wang et al., 2025; Chapa-Dubocq et al., 2023). The following sections summarize disease-associated phenotypes, the strength of available evidence, and potential therapeutic implications (Table 1).

**TABLE 1:** Reported alterations of CHCHD3 expression or function and associated mitochondrial phenotypes in disease models.

Disease category	Disease/model system	Reported CHCHD3 alteration	Major mitochondrial/pathological phenotypes	Evidence type	Key references
Neurodegenerative and neurological diseases	Alzheimer’s disease; AD patient brain tissue and 5xFAD transgenic mice	Reduced CHCHD3 protein abundance; stress-associated OMA1-dependent N-terminal processing has been reported in AD-related models	Cristae junction disorganization, impaired respiratory-chain supercomplex organization, ATP deficiency, cytochrome c mobilization, and increased neuronal apoptotic susceptibility	Human tissue association; transgenic mouse model; mechanistic validation in experimental systems	Tang et al. (2020a), Wang et al. (2025)
	Intracerebral hemorrhage; rat collagenase-induced ICH model	Increased total CHCHD3 abundance, possibly reflecting a compensatory response; functional destabilization of the SAM50–CHCHD3–MIC60 axis despite elevated protein level	Mitochondrial fragmentation, reduced membrane contact-site integrity, oxidative stress, neurological deficits; CHCHD3 overexpression partially restores axis stability and improves mitochondrial outcomes	Animal model; gain-of-function intervention	Yang et al. (2023)
	Cerebral ischemia–reperfusion injury; rat middle cerebral artery occlusion/reperfusion model	Functional disruption of the SAM50–CHCHD3 interaction; total CHCHD3 abundance may be initially preserved	Cristae disorganization, increased infarct volume, neuronal injury; SAM50 overexpression partially preserves axis integrity and mitigates mitochondrial damage	Animal I/R model; protein-interaction and rescue analyses	Yin et al. (2022)
	Postoperative cognitive dysfunction; aged rat surgery model	CHCHD3 reduction after electroacupuncture treatment relative to untreated surgical controls	Enhanced mitophagy, reduced ROS accumulation, decreased IL-1β production, improved spatial memory; CHCHD3 overexpression suppresses mitophagy and aggravates oxidative stress in this context	Animal model; intervention study; context-dependent quality-control mechanism	Zhang et al. (2024), Guo et al. (2023)
Cardiovascular diseases	Congenital heart disease/hypoplastic left heart syndrome; HLHS patient genomic studies and *Drosophila* cardiac-specific CHCHD3 RNAi	Candidate genetic association in HLHS; cardiac-specific CHCHD3 knockdown in *Drosophila*	Systolic dysfunction, sarcomeric protein depletion, ATP deficiency, and mitochondrial fission–fusion imbalance	Human genetic association; functional validation in *Drosophila*	Birker et al. (2023)
	Myocardial ischemia–reperfusion injury; murine and rat cardiac I/R models	OMA1-mediated N-terminal processing of CHCHD3, impairing CHCHD3 axis activity and potentially disrupting N-terminal lipidation-dependent mitochondrial association	MIB destabilization, cristae junction remodeling, respiratory-chain supercomplex disorganization, ROS accumulation, and cardiomyocyte apoptosis	Animal I/R models; mechanistic proteolysis and mitochondrial ultrastructure analyses	Tang et al. (2020b), Xue et al. (2019a)
	Cardiac aging; aged murine hearts examined by three-dimensional electron microscopy	Age-associated impairment of MICOS organization, potentially involving reduced CHCHD3-dependent cristae maintenance	Progressive cristae architectural deterioration, altered lipid metabolism, energetic insufficiency, and diastolic dysfunction	Aging mouse model; ultrastructural and metabolic analyses	Vue et al. (2023b)
	Metabolic cardiomyopathy; obscurin/obscurin-like 1 double-knockout murine hearts	Secondary reduction or destabilization of CHCHD3-associated mitochondrial organization following loss of cytoskeletal–mitochondrial coupling	Mitochondrial ultrastructural damage, diastolic dysfunction, and deregulated mitophagy	Genetic mouse model; cytoskeletal–mitochondrial coupling analysis	Xue et al. (2019a), Xue et al. (2019b)
Cancer	Hepatocellular carcinoma; HCC cell lines and clinical specimens	CHCHD3 upregulation; expression positively associated with tumor grade and invasive features	Increased mitochondrial ROS production, NF-κB activation, epithelial–mesenchymal transition-related changes, proliferation, migration, and invasion	Human tumor association; cell-based functional studies	Meng et al. (2024)
	Non-small cell lung cancer; NSCLC cell lines and patient-derived xenografts	CHCHD3 upregulation under hypoxic conditions, potentially through HIF-1α–USP3-dependent stabilization	Tumor growth, hypoxic adaptation, and poor prognosis-associated phenotypes	Cell models; xenografts; proposed ubiquitin-dependent stabilization mechanism	Zhao et al. (2026)
	Prostate cancer; prostate cancer cell lines and clinical samples	CHCHD3 upregulation associated with ASB1 E3 ligase downregulation and reduced K48-linked ubiquitination	Sustained CHCHD3/ROS-associated signaling, tumor cell proliferation, aggressive behavior, and potential therapy resistance	Human sample association; cell-based ubiquitination and functional assays	Zhao et al. (2024)
	Ferroptosis-sensitive tumors; RB1CC1-inducible cancer cell lines and xenograft models	Supraphysiological transcriptional induction of CHCHD3 through the RB1CC1–ELP3–H4K12ac axis	Mitochondrial membrane-potential hyperpolarization, excessive ROS accumulation, and ferroptotic cell death	Cell and xenograft models; transcriptional and ferroptosis mechanism analysis	Gong et al. (2025), Xue et al. (2022)
Metabolic diseases and reproductive-metabolic disorders	MASH/NASH; hepatocyte-specific CHCHD3 knockout mice and obese/high-fat diet models	Reduced CHCHD3 expression in metabolic stress models; genetic ablation in hepatocyte-specific knockout mice	Cristae disorganization, reduced ER–mitochondria contact sites, impaired lipid handling, hepatic steatosis, inflammation, and fibrosis-like pathology	Liver-specific knockout model; obese/diet-induced metabolic disease models	Vue et al. (2024b), Di Pasqua et al. (2025)
	Cardiac metabolic syndrome; high-fat diet-fed mice and db/db mice	Functional impairment of CHCHD3-associated cristae organization despite limited or variable changes in total CHCHD3 abundance	Reduced ATP synthesis, ROS accumulation, insulin resistance-associated mitochondrial dysfunction, and cardiomyopathy	Metabolic disease models; mitochondrial functional analyses	Xue et al. (2019a)
	PCOS-associated pregnancy loss; uterine decidua from early-pregnancy PCOS patients	Downregulation of cristae biogenesis-related genes, including CHCHD3	Abnormal mitochondrial ultrastructure, androgen excess-associated decidual metabolic defects, and impaired implantation-related tissue adaptation	Human tissue association; transcriptomic/ mitochondrial phenotype correlation	Zhang et al. (2026a)

### 6.1 Neurodegenerative diseases

Neurons are especially vulnerable to mitochondrial architectural defects because of their high dependence on oxidative phosphorylation and limited regenerative capacity (Darshi et al., 2011). Across neurological disease models, CHCHD3-associated injury converges on disruption of MICOS/MIB-dependent cristae organization, respiratory dysfunction, oxidative stress, and impaired mitochondrial quality control (Darshi et al., 2011).

Alzheimer’s disease (AD). CHCHD3 protein abundance is reduced by approximately 40%–50% in brains from patients with AD and in transgenic disease models, accompanied by weakening of the SAM50–CHCHD3–MIC60 axis, cristae disorganization, metabolic impairment, and neuronal apoptosis (Wang et al., 2025). Amyloid-β-associated stress further enhances OMA1-dependent CHCHD3 processing, thereby exacerbating MICOS/MIB destabilization (Tang et al., 2020a). Conversely, restoration of CHCHD3 expression attenuates amyloid-β-induced mitochondrial injury in experimental systems (Wang et al., 2025).

Intracerebral hemorrhage (ICH). In experimental ICH, total CHCHD3 abundance is increased, potentially as a compensatory response; however, hemorrhagic stress disrupts the functional integrity of the SAM50–CHCHD3–MIC60 axis despite this increase (Yang et al., 2023). CHCHD3 overexpression partially restores axis stability, improves mitochondrial function, and ameliorates neurological outcomes, indicating that preservation of complex integrity, rather than protein abundance alone, is critical for protection (Yang et al., 2023).

Seizure susceptibility. Neuron-specific deletion of CHCHD3 causes severe cristae disorganization, mitochondrial DNA release, and activation of cGAS–STING signaling, resulting in neuroinflammation and spontaneous seizures (He et al., 2022).

Cerebral ischemia–reperfusion injury. Ischemia–reperfusion disrupts SAM50–CHCHD3 interactions, leading to cristae disorganization and infarct aggravation; conversely, SAM50 overexpression partially preserves axis integrity and improves neuronal survival (Yin et al., 2022).

Postoperative cognitive dysfunction (POCD). In contrast to conditions in which CHCHD3 insufficiency is pathogenic, POCD may represent a setting in which maintenance of dysfunctional mitochondria becomes maladaptive (Zhang et al., 2024). In aged postoperative models, interventions that reduce CHCHD3 facilitate mitophagic clearance, whereas CHCHD3 overexpression suppresses mitophagy and exacerbates oxidative stress, highlighting the context dependence of CHCHD3-directed modulation (Guo et al., 2023).

### 6.2 Cardiovascular diseases

Congenital heart disease. Whole-genome sequencing of 183 families with hypoplastic left heart syndrome (HLHS) identified CHCHD3 as a candidate disease-associated gene (Birker et al., 2023). Consistently, cardiac-specific Chchd3 knockdown in *Drosophila* causes systolic dysfunction, depletion of sarcomeric proteins, ATP deficiency, and disturbed mitochondrial dynamics (Birker et al., 2023).

Myocardial ischemia–reperfusion injury. During ischemia–reperfusion, stress-activated OMA1 cleaves CHCHD3, thereby impairing the SAM50–CHCHD3–MIC60 axis and contributing to MIB destabilization, disorganization of respiratory-chain supercomplexes, ROS accumulation, and cardiomyocyte apoptosis (Tang et al., 2020b; Li et al., 2024). Preservation of CHCHD3 integrity, or experimental CHCHD3 overexpression, stabilizes MICOS/MIB architecture and attenuates ischemic mitochondrial injury (Darshi et al., 2011; Yin et al., 2022).

Cardiac aging and metabolic cardiomyopathy. Aged murine hearts display progressive cristae deterioration and energetic decline, potentially linked to impaired CHCHD3-dependent maintenance of MICOS integrity (Vue et al., 2023b). In metabolic cardiomyopathy, pyridostigmine indirectly improves CHCHD3-associated MICOS stability through activation of the LKB1/AMPK pathway, thereby ameliorating diastolic dysfunction (Xue et al., 2019a; Xue et al., 2019b).

### 6.3 Cancer

CHCHD3 exerts context-dependent effects in cancer by linking mitochondrial architecture to ROS signaling and cell-death susceptibility (Ding et al., 2025). Under basal or growth-promoting conditions, elevated CHCHD3 generally favors tumor progression. In hepatocellular carcinoma, CHCHD3 activates NF-κB signaling through mitochondrial ROS and promotes epithelial–mesenchymal transition (Meng et al., 2024). In non-small cell lung cancer, a hypoxia-inducible factor-1α (HIF-1α)–ubiquitin-specific protease 3 (USP3) axis stabilizes CHCHD3 under hypoxic conditions to enhance tumor cell survival (Zhao et al., 2026). In prostate cancer, downregulation of ASB1 leads to CHCHD3 accumulation and therapy resistance (Zhao et al., 2024). By contrast, under ferroptosis-inducing conditions, RB1-inducible coiled-coil 1 (RB1CC1, also known as CCRP1) and elongator acetyltransferase subunit 3 (ELP3) drive transcriptional upregulation of CHCHD3, promoting mitochondrial membrane hyperpolarization and ROS accumulation and thereby sensitizing tumor cells to ferroptotic death (Gong et al., 2025; Xue et al., 2022). These observations indicate that therapeutic strategies targeting CHCHD3 will likely require tumor-specific and stress-context stratification rather than uniform inhibition or activation.

### 6.4 Metabolic diseases

Hepatocyte-specific deletion of CHCHD3 induces spontaneous hepatic steatosis, inflammation, and fibrosis even in the absence of dietary challenge, accompanied by cristae disorganization and reduced endoplasmic reticulum–mitochondria contacts (Vue et al., 2024b). Notably, even partial suppression of CHCHD3 (∼30–40%) is sufficient to impair lipid transfer and trigger steatosis, indicating a dose-sensitive requirement for CHCHD3 in hepatic metabolic homeostasis. In contrast, the FXR agonist INT-787 restores CHCHD3 expression and improves cristae morphology under metabolic stress conditions (Di Pasqua et al., 2025).

Cardiac metabolic syndrome. High-fat feeding induces myocardial cristae disorganization and ATP depletion, whereas pyridostigmine-mediated activation of AMPK preserves CHCHD3-associated mitochondrial architecture and mitigates cardiomyopathy (Xue et al., 2019a).

Reproductive metabolic disorders. In polycystic ovary syndrome, reduced CHCHD3 expression and abnormal mitochondrial ultrastructure have been observed in the uterine decidua, potentially contributing to adverse pregnancy outcomes (Zhang et al., 2026a).

### 6.5 Threshold effects and the “Goldilocks” principle of CHCHD3 expression

Available evidence suggests that CHCHD3 function is governed by both dosage and biological context (Ding et al., 2025). Below a lower functional threshold, complete loss of CHCHD3 causes catastrophic cristae vesiculation and respiratory failure, whereas partial reduction—as reported in AD (∼40–50%) and metabolic stress models (∼30–40%)—results in progressive destabilization of the SAM50–CHCHD3–MIC60 axis and sublethal mitochondrial dysfunction (Funck, 2022; Tang et al., 2020b; Darshi et al., 2011; Darshi et al., 2012). At the other extreme, increased CHCHD3 expression may be adaptive or pathogenic depending on context: fasting-induced upregulation supports β-oxidation, whereas sustained elevation in cancer can facilitate ROS-dependent malignancy, excessive accumulation in POCD suppresses mitophagy, and RB1CC1–ELP3-driven induction during ferroptotic stress enhances cell death (Zhang et al., 2023; Sohn et al., 2023; Guo et al., 2023; Meng et al., 2024; Gong et al., 2025). This relationship is further complicated by the fact that total abundance does not necessarily reflect functional competence, as illustrated in ICH, where CHCHD3 levels increase despite collapse of axis integrity (Yang et al., 2023). Together, these observations support a “Goldilocks” model in which both insufficient and excessive CHCHD3 activity may be detrimental (Orhan et al., 2026). Therapeutic intervention should therefore be guided by disease-specific expression patterns, complex assembly status, and cellular stress context rather than by a uniform strategy of CHCHD3 augmentation or suppression (Zhang and Lou, 2025).

## 7 Therapeutic targeting of CHCHD3: opportunities and challenges

To date, no highly selective small molecule or biologic specifically targeting CHCHD3 has entered clinical development. Existing therapeutic approaches can be broadly divided into two categories: direct binders and indirect modulators (Table 2).

**TABLE 2:** Comparative evaluation of CHCHD3-targeting therapeutic strategies.

Molecule/Strategy	Mechanism of action	Target specificity	Stage of development	Key pharmacological limitations
Puerarin	Direct binding to CHCHD3; promotes proteasomal degradation	Moderate; multi-target flavonoid	Preclinical (*in vitro*; murine tumour models)	KD = 14.26 μM reported by SPR; kon/koff undefined; promiscuous target profile; poor water solubility and oral bioavailability; unclear selectivity among CHCHD paralogs
Pyridostigmine bromide	Indirect; LKB1/AMPK/ACC axis activation	Low; non-specific mitochondrial biogenesis	Preclinical (rodent metabolic syndrome models)	Not a direct CHCHD3 modulator; narrow therapeutic index due to systemic cholinergic effects; limited cardiac specificity
INT-787	Indirect; FXR agonism restoring CHCHD3 transcription	Low; nuclear receptor pleiotropy	Preclinical (rodent MASH/NASH models)	Hepatotoxicity and pruritus risk; broad metabolic transcriptional reprogramming beyond CHCHD3; species-specific FXR responses
CHCHD3 gene therapy (AAV)	Viral vector-mediated overexpression	High (theoretically); promoter-dependent	Early preclinical (neurological and cardiac models)	Irreversibility; lack of dose control; immune responses to viral vectors; tissue-specific delivery barriers; risk of supraphysiological expression
ASB1 activators (conceptual)	Promotion of K48-linked ubiquitination and CHCHD3 degradation	High (if allosteric specificity achieved)	Discovery stage	Not yet validated; risk of excessive CHCHD3 depletion causing cristae destruction and energetic failure
RB1CC1 (RB1-inducible coiled-coil 1)–ELP3-inducible cancer cell lines and xenograft models	Transcriptional upregulation for ferroptosis sensitization	Moderate; epigenetic axis specificity uncertain	Discovery stage	Tissue-specific toxicity; narrow therapeutic window between tumour ferroptosis and normal tissue oxidative injury
PROTACs/molecular glues (conceptual)	Induced CHCHD3 degradation via E3 ligase recruitment	High (if ligand-developed)	Discovery stage	Requires validated CHCHD3 ligand and ternary complex optimization; risk of excessive degradation

Direct binders. Puerarin, an isoflavone isolated from Pueraria lobata, is currently the only reported compound with direct interaction with CHCHD3 (102). Drug affinity responsive target stability (DARTS) and cellular thermal shift assay (CETSA) analyses suggest that puerarin promotes proteasomal degradation of CHCHD3 in tumor-infiltrating regulatory T cells (Li et al., 2026). However, its pharmacological characterization remains incomplete. Although surface plasmon resonance (SPR) analysis indicates a moderate binding affinity (K_D ≈ 14.26 μM), the precise binding interface and structural basis of target engagement have not been defined, and selectivity across CHCH domain-containing paralogs, including CHCHD6/MIC25 and CHCHD10/MIC14, has not been established (Li et al., 2026). Moreover, because puerarin is a pleiotropic flavonoid with reported activity toward the estrogen receptor, PI3K/Akt signaling, and multiple redox-sensitive pathways, its target attribution and *in vivo* specificity remain uncertain (Wang et al., 2022).

Indirect modulators. Several compounds influence CHCHD3-associated mitochondrial phenotypes without directly binding CHCHD3. In models of metabolic dysfunction-associated steatohepatitis, the FXR agonist INT-787 reverses high-fat diet-induced downregulation of CHCHD3 and improves cristae morphology and ATP production (Di Pasqua et al., 2025). However, FXR controls extensive transcriptional programs related to bile acid metabolism, lipid turnover, glucose homeostasis, and inflammation, making it difficult to distinguish CHCHD3-dependent effects from broader systemic responses (Huang et al., 2022; Ding et al., 2015; Calkin and Tontonoz, 2012). In addition, FXR agonism is associated with adverse effects such as pruritus and hepatobiliary toxicity, and the therapeutic window required to restore CHCHD3 without provoking systemic metabolic disturbance remains undefined (Fiorucci et al., 2025; Cho and Lee, 2022). Likewise, pyridostigmine improves cardiac cristae integrity and diastolic function in metabolic syndrome models through activation of the LKB1/AMPK/ACC signaling axis (Xue et al., 2019a). As pyridostigmine is not a CHCHD3 ligand, its effects on the CHCHD3–MICOS pathway are indirect and secondary (Xue et al., 2019a). Its translational utility may also be limited by systemic cholinergic adverse effects, including bradycardia and bronchospasm, particularly in older patients or those with cardiopulmonary comorbidities.

Gene-based strategies. Adeno-associated virus (AAV)-mediated CHCHD3 overexpression has shown beneficial effects in preclinical models of Alzheimer’s disease, intracerebral hemorrhage, and cardiac ischemia–reperfusion injury. Nevertheless, sustained transgene expression raises important concerns given the apparent dose-sensitive biology of CHCHD3. Supra-physiological expression may prove maladaptive in certain settings by suppressing mitophagy, aggravating oxidative stress, or increasing susceptibility of non-malignant tissues to ferroptotic injury. Additional barriers to translation include vector immunogenicity, incomplete tissue specificity, and off-target transduction (Park-Wyllie et al., 2009; Broad et al., 2013).

Future directions. Further progress will require at least three advances. First, direct CHCHD3 ligands with rigorously validated binding kinetics, structurally defined interaction sites, and selectivity across the CHCH protein family are needed. Second, therapeutic strategies should be designed to preserve or restore CHCHD3 function within its physiological range, with particular attention to mitochondrial localization, incorporation into MICOS/MIB complexes, and redox-dependent structural integrity rather than simple modulation of total protein abundance. Third, robust target-engagement and pharmacodynamic biomarkers—such as CHCHD3 occupancy assays or quantitative readouts of cristae junction integrity—will be essential, ideally in patient-derived cellular systems or organoids (Gottschalk et al., 2019; Liang, 2024). More broadly, successful therapeutic development will likely depend on a precision-based framework integrating disease stage, CHCHD3 expression status, and mitochondrial functional state in order to maximize efficacy while minimizing unintended cellular stress (Arena et al., 2026).

## 8 Conclusion and perspectives

CHCHD3 serves as a central determinant of mitochondrial cristae architecture by linking structural support within the MICOS and MIB complexes to stress-responsive regulatory mechanisms, including myristoylation, phosphorylation, ubiquitination, and proteolytic processing (Tang et al., 2020b; Darshi et al., 2011; Darshi et al., 2012). Disruption of CHCHD3 expression, submitochondrial localization, or functional integrity has been implicated in a wide range of human disorders, including neurodegenerative, cardiovascular, metabolic, and neoplastic diseases (Darshi et al., 2012). To date, therapeutic strategies targeting CHCHD3—including the direct binder puerarin, the indirect modulators INT-787 and pyridostigmine, and AAV-based gene delivery—remain at the preclinical stage and are limited by insufficient target specificity, pleiotropic off-target effects, or poor dose controllability (Xue et al., 2019a; Di Pasqua et al., 2025; Li et al., 2026) (see Section 7). Moreover, the narrow functional range suggested by the CHCHD3 “Goldilocks” principle indicates that future interventions will need to be precisely tailored to disease context rather than based on uniform activation or inhibition.

Several unresolved questions continue to define the field. First, the upstream pathways that coordinate CHCHD3 transcription, protein turnover, and post-translational modification under physiological and pathological conditions remain only partially characterized (Tang et al., 2020b; Darshi et al., 2012). Second, CHCHD3 exhibits marked context dependence. It appears protective in Alzheimer’s disease and intracerebral hemorrhage, whereas excessive accumulation may impair mitophagy in postoperative cognitive dysfunction and promote tumor progression through ROS–NF-κB signaling; under ferroptotic stress, however, CHCHD3 induction can instead enhance cell death susceptibility (Xue et al., 2022). The molecular basis underlying these divergent effects remains unclear. Third, the dynamic remodeling of the SAM50–CHCHD3–MIC60 axis during OMA1-dependent stress responses has been difficult to resolve in living cells with adequate spatial and temporal precision (Kondadi et al., 2020).

Addressing these issues will require advances at both the mechanistic and translational levels (Ott et al., 2015; Wang et al., 2025). High-resolution structural analysis of CHCHD3 in complex with MIC60, SAM50, and stress-responsive regulatory partners will be essential for defining interaction interfaces and regulatory conformations (Pape et al., 2020). Complementary live-cell super-resolution imaging and single-molecule approaches should help delineate the kinetics of MICOS/MIB remodeling under stress (Lin et al., 2023; Zhang et al., 2026b). In parallel, spatially resolved multi-omics will be needed to construct systems-level maps of CHCHD3-associated pathways across tissues and disease states (Lee et al., 2021; Garranzo-Asensio et al., 2020; Lv et al., 2022). From a clinical perspective, well-designed multicenter prospective studies are required to determine whether CHCHD3 abundance, submitochondrial distribution, or pathway-associated signatures have diagnostic, prognostic, or treatment-stratification value (Wang et al., 2025). At present, no CHCHD3-specific therapeutic has entered clinical evaluation, underscoring the need for systematic preclinical assessment of pharmacokinetics, tissue selectivity, and off-target liability.

Looking ahead, three areas merit particular priority. First, structure-guided development of selective CHCHD3 modulators—including both stabilizers and targeted degraders—should be pursued on the basis of orthosteric or allosteric pockets defined by high-resolution structural studies (Darshi et al., 2011; Darshi et al., 2012). Second, comprehensive mapping of CHCHD3 post-translational regulation should be extended beyond currently recognized modifications to include acetylation, O-GlcNAcylation, and other emerging marks, with particular attention to their functional interplay (Darshi et al., 2011; Prisco et al., 2020; Adaniya et al., 2019; McLarty et al., 2013; Morales and Pratt, 2024). Third, biomarker-driven translational studies are needed to evaluate CHCHD3-associated signatures for patient stratification and therapeutic monitoring (Ježek et al., 2023; Darshi et al., 2011). Given that CHCHD3 operates within broader mitochondrial quality-control and redox networks, combination strategies may ultimately prove more practical than CHCHD3-directed monotherapy, particularly in settings such as ferroptosis sensitization in cancer, FXR-based intervention in MASH, and protection against ischemia–reperfusion injury in cardiovascular disease (Ježek et al., 2023; Di Pasqua et al., 2025; Balasco et al., 2025; Liu et al., 2023; Lillo-Moya et al., 2021). With continued advances in structural biology, mitochondrial pharmacology, and disease-stratified investigation, CHCHD3 is likely to emerge as an important node for mechanism-informed biomarker discovery and therapeutic development (Darshi et al., 2012; Balasco et al., 2025).
